# A Higher-Protein, Energy Restriction Diet Containing 4 Servings of Fresh, Lean Beef per Day Does Not Negatively Influence Circulating miRNAs Associated with Cardiometabolic Disease Risk in Women with Overweight

**DOI:** 10.1016/j.cdnut.2024.104442

**Published:** 2024-08-18

**Authors:** Kamille A Piacquadio, Jess A Gwin, Heather J Leidy

**Affiliations:** 1 Department of Nutritional Sciences and Department of Pediatrics; University of Texas at Austin; Austin, TX, United States; 2 Military Nutrition Division, U.S. Army Research Institute of Environmental Medicine, Natick, MA, United States

**Keywords:** red meat, miRNA expression, negative energy balance, cardiometabolic health, protein quantity

## Abstract

This study examined the acute effects of 7-d energy restriction normal-protein (NP; ∼15% of daily intake as protein) compared with higher-protein (HP; ∼38% of daily intake as protein) diets varying in quantities of fresh, lean beef on circulating miRNA expression associated with cardiometabolic disease in 16 women with overweight (mean ± SD; age: 35 ± 8.7 y; body mass index: 28.5 ± 1.9 kg/m^2^). Fasting blood samples were collected at the end of each diet for miRNA expression, glucose, insulin, adiponectin, C-reactive protein (CRP), and IL-6. Of the 12 surveyed, 10 miRNAs (miR-320a-3p, miR-146a-5p, miR-150-5p, miR-423-5p, miR-122-5p, miR-223-3p, miR-199a-5p, miR-214-3p, miR-24-3p, and miR-126-3p) were detected. Several miRNAs were associated with fasting CRP (i.e., miR-150-5p, miR-24-3p, miR-423-5p; all *P* < 0.05). miR-423-5p was also associated with fasting glucose, IL-6, and homeostasis model assessment 2 %β cell function (all, *P* < 0.05). No differences in miRNA expression were identified between diets. These data suggest that fresh, lean beef in a short-term HP, energy restriction diet does not negatively influence circulating miRNAs associated with cardiometabolic disease in women.

This trial was registered at clinicaltrials.gov as NCT02614729.

## Introduction

In the United States, over 50% of adults have ≥1 chronic health condition with 30% experiencing multiple conditions, all of which reduce quality of life, life expectancy, and increase health care costs [1,2]. Obesity is a leading cause of preventable death in the United States and is an independent risk factor for metabolic diseases including type 2 diabetes (T2D) and cardiovascular disease (CVD). Emerging regulators identified in the development and progression of chronic, metabolic diseases are a family of small (∼22 nucleotides), noncoding RNA molecules known as microRNAs (miRNAs) that modulate gene expression at the posttranscriptional level [3]. Triggered by local stimuli, including processes related to metabolic dysfunction (i.e., inflammation), miRNAs are released into circulation and regulate expression of genes in the same or in distal tissues including adipose tissue, pancreas, liver and muscle [4]. Further, when comparing individuals who were metabolically healthy with those with a chronic condition (obesity, T2D), circulating miRNA profiles varied dependent on health status [5]. Thus, aberrant expression of these posttranscriptional modifiers, as a result of excessive adiposity, contributes to sustained metabolic dysfunction [[6], [7], [8]]. Although the relationship between chronic disease and miRNA expression continues to develop, the influence of dietary factors remains relatively unknown.

The most common dietary strategy promoted to combat obesity and reduce cardiometabolic disease risk includes energy restriction diets ranging from −500 to −750 kcal/d [9,10]. However, the ability to adhere to energy restriction diets over the longer term is challenging because of increased hunger and food cravings along with blunted satiety [10,11]. Thus, 1 strategy to improve appetite control and satiety during energy restriction is through increasing dietary protein intake [12,13]. Additional benefits of higher-protein (HP) diets (containing 27%–35% of daily energy as protein) include greater reductions in body weight and fat mass compared with standard protein diets (containing 16%–21% of energy as protein) as highlighted by a meta-analysis performed by Wycherley et al. [14]. The majority of HP, energy restriction randomized controlled trials include animal-source protein-rich foods, particularly red meat, as part of the HP comparison [15,16]. Observational evidence suggests that red meat consumption is highly correlated with increased risk of developing obesity, T2D, CVD, some cancers, and all-cause mortality [17]. As such, the 2020–2025 Dietary Guidelines for Americans [17] emphasize healthy dietary patterns that include plant-based foods but limit the consumption of red and processed meats. However, a number of intervention-based trials evaluated within published meta-analyses [18,19] challenge these findings. Further, although fresh lean beef as part of a healthy pattern has been shown to be beneficial for CVD risk factors, total cholesterol, and LDL cholesterol [20], it is unclear as to whether these effects occur at the molecular level via changes in miRNA expression.

The purpose of this study was to identify whether an energy restriction, HP diet, containing ∼4 servings of fresh, lean beef per day alters expression of circulating miRNAs selected based on their recorded signatures in obesity, T2D and CVD compared with an energy restriction, normal-protein (NP) diet containing ∼1 serving of fresh, lean beef.

## Methods

### Experimental design

Secondary analyses were performed from an acute crossover design study in 16 women with overweight with no chronic conditions or diseases. The purpose of the original study was to examine the effects of NP compared with HP, energy restriction (−750 kcal/d) diets on appetite control, satiety, and ad libitum intake [21]. Fasting blood samples were collected at the end of each dietary pattern for analysis of appetite and satiety hormones. In this analyses, fasting blood samples were analyzed for miRNA expression and markers of cardiometabolic disease risk. The original trial design is reported at clinicaltrials.gov as NCT02614729.

### Study participants

From January 2014 to May 2015, women with overweight were recruited from the Columbia, MO, area through advertisements, flyers, and e-mail listservs to participate in the study. Seventeen women signed the consent, began, and completed the study. Of those, 16 participants were included in this secondary analyses based on availability of fasting blood samples. In general, participants were adult women with overweight (mean ± SD; age: 35 ± 8.7 y; BMI: 28.5 ± 1.9 kg/m^2^) with no chronic conditions or diseases. The majority (92%) were White and non-Hispanic/non-Latino. All participants were informed of the study purpose, procedures, and risks and signed the consent/assent forms. The study was approved by the University of Missouri Health Sciences institutional review board, and all procedures were followed in accordance with the ethical standards of the institutional review board. The participants received a stipend for completing all study procedures.

### Diet interventions

For 7 d/diet, the participants were provided with isocaloric, energy-restricted (−750 kcal) diets as either NP or HP. The NP diet included 1240 ± 0 kcal/d (%daily energy: 15.5% protein; 55.5% carbohydrates; 31.2% fat), whereas the HP diet included 1280 ± 10 kcal/d (%daily energy: 38.8% protein; 39% carbohydrates; 23.2% fat). The sources of protein were similar across diets and consisted of fresh, lean beef (60% of total protein) as flank and top round steak and plant proteins (40% of total protein) as textured soy protein, tofu, and wheat gluten products. The NP diet contained ∼1 serving/d of fresh, lean beef (4 ounces/d), whereas the HP diet contained ∼4 servings/d of fresh, lean beef (15 ounces/d).

### Fasting blood sampling and analyses

Blood samples (4 mL/sample) were collected after an overnight fast at the end of each 7-d period. Collection, processing, and storage methods are published elsewhere [22].

The plasma fasting markers were measured as follows: insulin (Ultrasensitive Insulin ELISA; ALPCO), C-reactive protein (C-Reactive Protein ELISA; ALPCO), adiponectin (Human Adiponectin ELISA; Millipore), IL-6 (Human IL-6 High Sensitivity ELISA; Invitrogen), and glucose (colorimetric assay; Cayman Chemical).

Total RNA extraction with UniSp2 spike-in, cDNA synthesis, miRNA quantification, and sample hemolysis assessment methodologies are published elsewhere [22]. There were no differences detected in miRNA spike-in, UniSp2 between treatments (C_T_ values mean ± SD: NP, 14.22 ± 0.80; HP: 14.40 ± 0.96). Expression levels of miR-21-5p, miR-320a-3p, miR-146a-5p, miR-150-5p, miR-423-5p, miR-15b-5p, miR-122-5p, miR-223-3p, miR-199a-5p, miR-214-3p, miR-24-3p, and miR-126-3p were normalized to the geometric mean of (external control) UniSp2 and (internal control) SNORD44 (C_T_ values, mean ± SD, CV—NP: 20.89 ± 1.03, 4.93%; HP: 21.28 ± 1.13, 5.29%). The ΔΔC_T_ method was used for quantification of miRNA and relative expression is reported as 2^−ΔΔCT^. Fold change data were expressed relative to mean NP values. miR ratios for hemolysis assessment for NP (mean ± SD, 2.77 ± 0.79) and HP (mean ± SD, 3.12 ± 0.71) indicated that hemolysis did not influence any differences observed between treatment groups after filtering out 3 samples and their associated pair due to experimental design of the study. This reduced total sample size for quantification of miRNA expression (*n* = 13).

### Data and statistical analyses

Power analyses were performed from data generated by a study evaluating dietary intervention influence on circulating miRNA expression [23]. A sample size of 9 was suggested to provide 80% power to detect meaningful differences in expression from the mean differential of 3.98 (fold change) and SD of difference of 3.56, an effect size of 1.12. β-Cell function, insulin resistance and sensitivity were examined via homeostasis model assessment (HOMA) 2-insulin resistance, HOMA2 β-cell function (%), and HOMA2-insulin sensitivity (%) calculations (https://www.dtu.ox.ac.uk/homacalculator/).

Summary statistics were generated and normality assessment of the data via Shapiro–Wilk test was performed before statistical testing. Outliers, identified as data points falling outside 1.5 times the interquartile range below Q1 or above Q3 were removed from the data set. All miRNA data were log-transformed (log_2_) for statistical analyses. Paired-sample *t* tests were performed to evaluate the impact of the quantity of beef consumption during energy restriction on miRNA expression and markers of acute inflammation and glycemic control. If paired samples did not cross the C_T_ in both NP and HP diets, these samples were excluded from the analyses.

Pearson correlational analyses were performed to identify associations between the expression of circulating miRNAs and fasting cardiometabolic biomarkers. Each statistical test was performed using *P* ≤ 0.05 as criterion for statistical significance. Statistical analyses were performed in IBM SPSS Statistics (version: 28.0.0.0).

## Results

**FIGURE 1:**
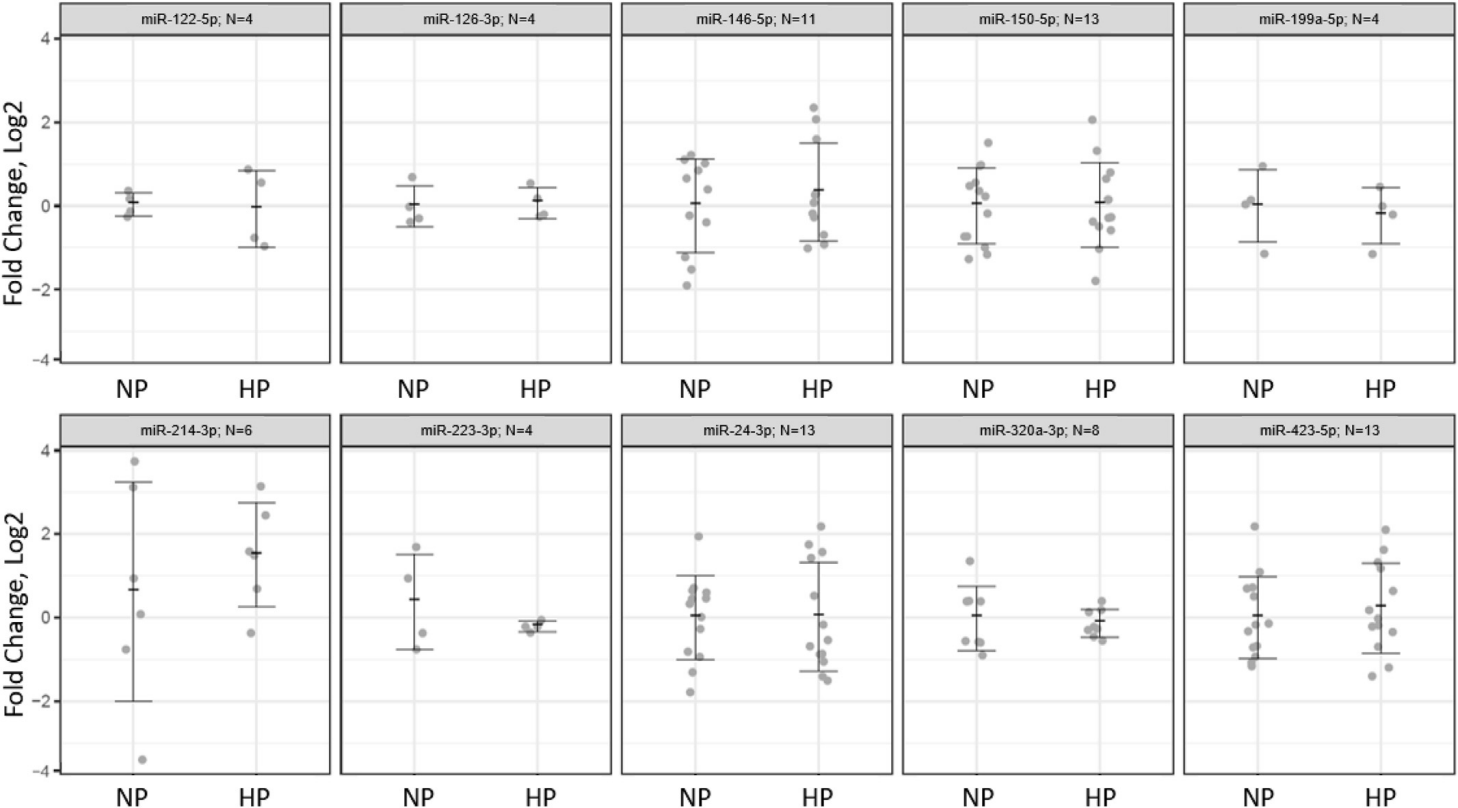
Dot plots of circulating miRNA for comparison between the normal-protein (NP) and higher-protein (HP) diets via paired-sample *t* tests. Values are means ± SD.

A total of 10 of the 12 a priori miRNAs were detected following both diets as shown in Figure 1. No differences in the expression of miRNAs surveyed were observed between the NP and HP diets. Correlational analyses performed to identify miRNA-cardiometabolic marker associations are reported in Table 1. A number of miRNAs were positively associated with fasting C-reactive protein (i.e., miR-150-5p, miR-24-3p, miR-423-5p; all *P* < 0.05). Further, miR-423-5p was inversely associated with fasting glucose and positively associated with IL-6 and HOMA2-β cell function (all *P* < 0.05).

**TABLE 1:** Pearson correlations between the relative expression of circulating miRNA and fasting cardiometabolic markers analyzed after the consumption of the normal-protein (NP) and higher-protein (HP) diets.

Variables	All		NP		HP	
	Correlation coefficient	*P*	Correlation coefficient	*P*	Correlation coefficient	*P*
miR-146-5p						
Adiponectin (μg/mL)	0.098	0.656	−0.043	0.901	0.268	0.400
Glucose (mg/dL)	−0.217	0.32	0.026	0.94	−0.460	0.132
CRP (mg/dL)	0.278	0.199	0.376	0.254	0.205	0.523
Insulin (pmol/L)	−0.15	0.493	0.118	0.73	−0.414	0.181
IL-6 (pg/mL)	−0.113	0.606	0.202	0.552	−0.396	0.202
HOMA2-IR	−0.159	0.468	0.109	0.751	−0.425	0.169
HOMA2-%B	−0.027	0.903	0.176	0.604	−0.248	0.436
HOMA2-%S	−0.056	0.799	−0.369	0.264	0.509	0.091
miR-150-5p						
Adiponectin (μg/mL)	−0.225	0.269	0.051	0.868	−0.534	0.06
Glucose (mg/dL)	−0.246	0.225	−0.013	0.966	−0.448	0.017^1^
CRP (mg/dL)	0.682	0.000^1^	0.653	0.015^1^	0.615	0.025^1^
Insulin (pmol/L)	0.26	0.200	0.169	0.580	0.141	0.645
IL-6 (pg/mL)	0.269	0.184	0.381	0.199	0.164	0.592
HOMA2-IR	0.245	0.227	0.166	0.588	0.108	0.726
HOMA2-%B	0.319	0.112	0.126	0.681	0.404	0.171
HOMA2-%S	−0.084	0.684	−0.059	0.849	0.047	0.878
miR-24-3p						
Adiponectin (μg/mL)	−0.154	0.453	−0.19	0.534	−0.127	0.680
Glucose (mg/dL)	−0.346	0.083	−0.145	0.637	−0.498	0.083
CRP (mg/dL)	0.607	0.001^1^	0.773	0.002^1^	0.585	0.036^1^
Insulin (pmol/L)	0.131	0.524	0.255	0.401	0.056	0.856
IL-6 (pg/mL)	0.251	0.216	0.597	0.031^1^	−0.022	0.943
HOMA2-IR	0.112	0.585	0.24	0.429	0.034	0.913
HOMA2-%B	0.261	0.197	0.32	0.287	0.228	0.454
HOMA2-%S	−0.056	0.785	−0.277	0.455	0.154	0.615
miR-423-5p						
Adiponectin (μg/mL)	−0.258	0.202	−0.262	0.387	−0.258	0.395
Glucose (mg/dL)	−0.42	0.033^1^	−0.177	0.564	−0.646	0.017^1^
CRP (mg/dL)	0.614	0.001^1^	0.682	0.01^1^	0.615	0.025^1^
Insulin (pmol/L)	0.232	0.254	0.315	0.295	0.141	0.645
IL-6 (pg/mL)	0.408	0.038^1^	0.681	0.01^1^	0.164	0.592
HOMA2-IR	0.207	0.310	0.298	0.323	0.108	0.726
HOMA2-%B	0.413	0.036^1^	0.397	0.179	0.404	0.171
HOMA2-%S	−0.195	0.341	−0.333	0.266	0.047	0.878
miR-320a-3p						
Adiponectin (μg/mL)	−0.044	0.856	−0.060	0.860	−0.002	0.996
Glucose (mg/dL)	−0.052	0.828	0.084	0.806	−0.396	0.292
CRP (mg/dL)	0.288	0.218	0.374	0.258	0.419	0.262
Insulin (pmol/L)	0.144	0.544	0.193	0.569	0.209	0.589
IL-6 (pg/mL)	0.090	0.706	0.240	0.477	−0.260	0.500
HOMA2-IR	0.138	0.560	0.189	0.578	0.196	0.613
HOMA2-%B	0.195	0.410	0.205	0.546	0.305	0.426
HOMA2-%S	−0.380	0.098	−0.457	0.158	−0.202	0.602
miR-126-3p						
Adiponectin (μg/mL)	0.032	0.926	0.410	0.590	0.037	0.938
Glucose (mg/dL)	−0.475	0.140	−0.404	0.596	−0.574	0.178
CRP (mg/dL)	0.671	0.024^1^	0.941	0.059	0.603	0.152
Insulin (pmol/L)	0.200	0.555	−0.067	0.933	0.137	0.770
IL-6 (pg/mL)	0.352	0.288	−0.835	0.165	0.337	0.459
HOMA2-IR	0.172	0.614	−0.096	0.904	0.105	0.823
HOMA2-%B	0.447	0.168	0.184	0.816	0.527	0.224
HOMA2-%S	−0.260	0.440	−0.204	0.796	−0.198	0.670
miR-214-3p						
Adiponectin (μg/mL)	−0.215	0.644	−0.082	0.862	−0.319	0.402
Glucose (mg/dL)	−0.443	0.086	−0.374	0.409	−0.661	0.052
CRP (mg/dL)	0.062	0.820	−0.519	0.233	0.320	0.401
Insulin (pmol/L)	0.117	0.666	−0.045	0.924	0.222	0.567
IL-6 (pg/mL)	0.028	0.918	−0.511	0.241	0.289	0.451
HOMA2-IR	0.087	0.749	−0.080	0.865	0.192	0.620
HOMA2-%B	0.338	0.201	0.267	0.563	0.451	0.223
HOMA2-%S	−0.113	0.678	0.023	0.961	−0.259	0.500
miR-199a-5p						
Adiponectin (μg/mL)	0.603	0.113	0.500	0.500	0.733	0.267
Glucose (mg/dL)	−0.590	0.124	−0.631	0.369	−0.540	0.460
CRP (mg/dL)	0.188	0.655	0.731	0.269	0.027	0.973
Insulin (pmol/L)	−0.657	0.077	−0.463	0.537	−0.904	0.096
IL-6 (pg/mL)	−0.477	0.232	−0.457	0.543	−0.588	0.412
HOMA2-IR	−0.698	0.054	−0.514	0.486	−0.932	0.068
HOMA2-%B	−0.125	0.767	0.039	0.961	−0.384	0.616
HOMA2-%S	0.447	0.267	0.234	0.766	0.880	0.120
miR-223-3p						
Adiponectin (μg/mL)	0.089	0.783	0.058	0.901	0.130	0.834
Glucose (mg/dL)	−0.426	0.168	−0.321	0.482	−0.655	0.231
CRP (mg/dL)	0.599	0.040^1^	0.013	0.978	0.879	0.049^1^
Insulin (pmol/L)	−0.013	0.969	−0.003	0.995	−0.066	0.916
IL-6 (pg/mL)	0.534	0.073	0.341	0.455	0.658	0.227
HOMA2-IR	−0.047	0.885	−0.031	0.948	−0.112	0.858
HOMA2-%B	0.355	0.258	0.258	0.576	0.633	0.252
HOMA2-%S	−0.191	0.552	−0.224	0.629	−0.062	0.922
miR-122-5p						
Adiponectin (μg/mL)	−0.128	0.691	0.126	0.812	−0.314	0.545
Glucose (mg/dL)	−0.318	0.314	−0.338	0.512	−0.345	0.504
CRP (mg/dL)	0.784	0.003^1^	0.755	0.083	0.812	0.050^1^
Insulin (pmol/L)	0.215	0.502	−0.050	0.926	0.407	0.423
IL-6 (pg/mL)	0.324	0.305	0.561	0.247	0.249	0.634
HOMA2-IR	0.193	0.547	−0.065	0.903	0.378	0.460
HOMA2-%B	0.437	0.155	0.225	0.669	0.604	0.204
HOMA2-%S	−0.164	0.610	−0.048	0.929	−0.537	0.272

Abbreviations: CRP, C-reactive protein; HOMA2-IR, homeostatic model assessment insulin resistance; HOMA2-%B, homeostatic model assessment β-cell function; HOMA2-%S, homeostatic model assessment insulin sensitivity.

1 *P* < 0.05.

## Discussion

Despite the varying protein quantity between these 7-d energy restriction diets (difference of: 76 g protein/d), no differences in miRNA expression were detected in women with overweight with no chronic conditions or diseases. These data suggest that the acute consumption of 4 servings of fresh, lean beef in a HP, energy restriction diet does not negatively influence miRNAs identified as potential mediators of cardiometabolic disease risk.

A limited number of intervention-based studies exist examining whether dietary factors alter CVD risk via circulating miRNA expression [24]. Of those that involve energy restriction, these studies vary in duration and dietary approach to evaluate the impact of glycemic index and load [25], macronutrient compositions [26], exercise [[27], [28], [29]], and meal replacements [30] on miRNA expression. Parr et al. [27] compared 16-wk diets varying in dairy protein and carbohydrates within an exercise-induced energy restriction on circulating miRNAs similar to those in this study. All interventions led to significant weight loss, which was accompanied by an increased expression of miR-223-3p, a suggested biomarker for obesity. We did not detect differences in miR-223-3p in this study for a number of potential reasons. The 7-d intervention design in this study limited our ability to assess weight loss. In addition, we included protein-rich foods from beef, whereas Parr et al. [27] included dairy; the differences in protein source may have also contributed to variable findings.

A recent systematic review of dietary impact on miRNA regulation [24] highlights that specific dietary factors (e.g., unsaturated fatty acids, plant-based foods) can regulate expression of the same miRNA differently. For example, in our previous study, we found that miR-15b-5p expression was higher after 7 d of consuming an energy balance diet containing fresh, lean beef compared with a diet void of fresh, lean beef [22]. The lack of difference in the panel of miRNAs surveyed in this study compared with other studies could be explained by the varying dietary factors, including protein quality and energy balance, across studies.

There are limitations to highlight, most notably that not all the miRNAs surveyed consistently crossed the C_T_, which resulted in a variable sample size across the analysis. We achieved ≥30% detection of miRNAs surveyed in the total sample, which is above the 20% cutoff incorporated in most studies [26]. Regardless, we want to mention several potential reasons for this occurrence. First, a single miRNA can be differentially expressed across different dietary patterns and across the magnitude of response to the intervention [24]. In addition, miRNA expression can vary between individuals based on whether those individuals are sensitive and responsive to the dietary interventions [26,27]. Finally, although sample collection can also impact the ability to detect miRNA expression in plasma, the samples collected in this study were stored and processed appropriately to detect circulating miRNAs. Additionally, baseline samples were not collected in this study, and thus, no preanalyses/postanalyses were available. The intervention was also short in duration (7 d) and is thus not generalizable to long-term changes. Although adequately powered, the sample size (*N* = 16) was relatively small with a fairly homogenous population of healthy women with overweight. Therefore, long-term randomized controlled trials with premeasurements and postmeasurements are needed to elucidate the impact of higher protein consumption, varying in protein quality and/or energy status, on a larger panel of miRNAs involved in cardiometabolic disease risk and their associated gene targets.

Collectively, our findings suggest that including fresh, lean beef in a short-term higher protein, healthy dietary pattern during energy restriction does not negatively influence circulating miRNAs associated with cardiometabolic disease development.

## Author contributions

The authors’ responsibilities were as follows – KAP, JAG, HJL: developed the research question; KAP: developed the associated methodology; KAP: performed the statistical analyses and interpretation of the data; KAP: wrote the first draft of the manuscript; KAP, HJL: had primary responsibility for the final content; and all authors: read and approved the final manuscript.

## Conflict of interest

KAP and JAG have no conflicts of interest to report. HJL is on the Editorial Board for the *Journal of Nutrition*. The opinions or assertions contained herein are the private views of the authors and are not to be construed as official or as reflecting the views of the Army or the Department of Defense. Any citations of commercial organizations and trade names in this report do not constitute an official Department of the Army endorsement or approval of the products or services of these organizations.

## Funding

The Beef Checkoff supplied the funds to complete the study but was not involved in the design, implementation, analyses or interpretation of the data.

## Data availability

Data described in the manuscript are available on request from the corresponding author.
